# Chromosome-level genome of the bank vole (*Clethrionomys glareolus*): a resource for eco-evo-disease research

**DOI:** 10.1038/s41597-026-06924-x

**Published:** 2026-02-26

**Authors:** Silvia Marková, Thomas A. White, Jeremy B. Searle, Petr Kotlík

**Affiliations:** 1 Laboratory of Molecular Ecology, Institute of Animal Physiology and Genetics, Czech Academy of Sciences, Liběchov, Czech Republic; 2 Department of Ecology and Evolutionary Biology, Cornell University, Ithaca, NY USA

**Keywords:** Evolutionary genetics, Ecological epidemiology, Climate-change ecology, Zoology

## Abstract

The increasing availability of reference genomes for non-traditional model species is enhancing research in ecology and evolution. Here, we present a high-quality chromosome-level genome assembly and annotation for the bank vole *Clethrionomys glareolus*, an emerging model mammal. The 2.24 Gb assembly is resolved into 28 chromosome-scale scaffolds, consistent with the known karyotype of the species. We predicted 40,393 gene loci, including 21,029 protein-coding genes supported by Swiss-Prot homology. Repetitive elements account for approximately 29% of the genome. The assembly achieves a 90.6% BUSCO score, underscoring its completeness. Compared to previously available fragmented assemblies, this genome enables investigation of genome architecture, selection and structural variation at unprecedented resolution. In particular, this chromosome-level resource supports research into ecological and evolutionary responses to climate variation across space and time. It also holds value for other fields, including developmental biology and studies of immunology and virology, where the bank vole is used as a model for host-pathogen interactions–expanding the relevance of this genomic resource across biological disciplines.

## Background & Summary

The increasing accessibility of high-quality reference genomes for non-traditional model species–facilitated by declining DNA sequencing costs–is rapidly advancing our ability to explore key questions in biology, particularly in ecology and evolution, with a level of precision once limited to major model organisms^1–3^. A major advantage of working with non-traditional species is the ability to select taxa based on ecological or evolutionary relevance, rather than on their long-standing use and convenience in laboratory settings^4–6^. Within this framework, we present the first chromosome-level genome assembly of the bank vole *Clethrionomys glareolus* (syn. *Myodes glareolus*^7^), an emerging model species increasingly used across multiple biological disciplines.

**Fig. 1:**
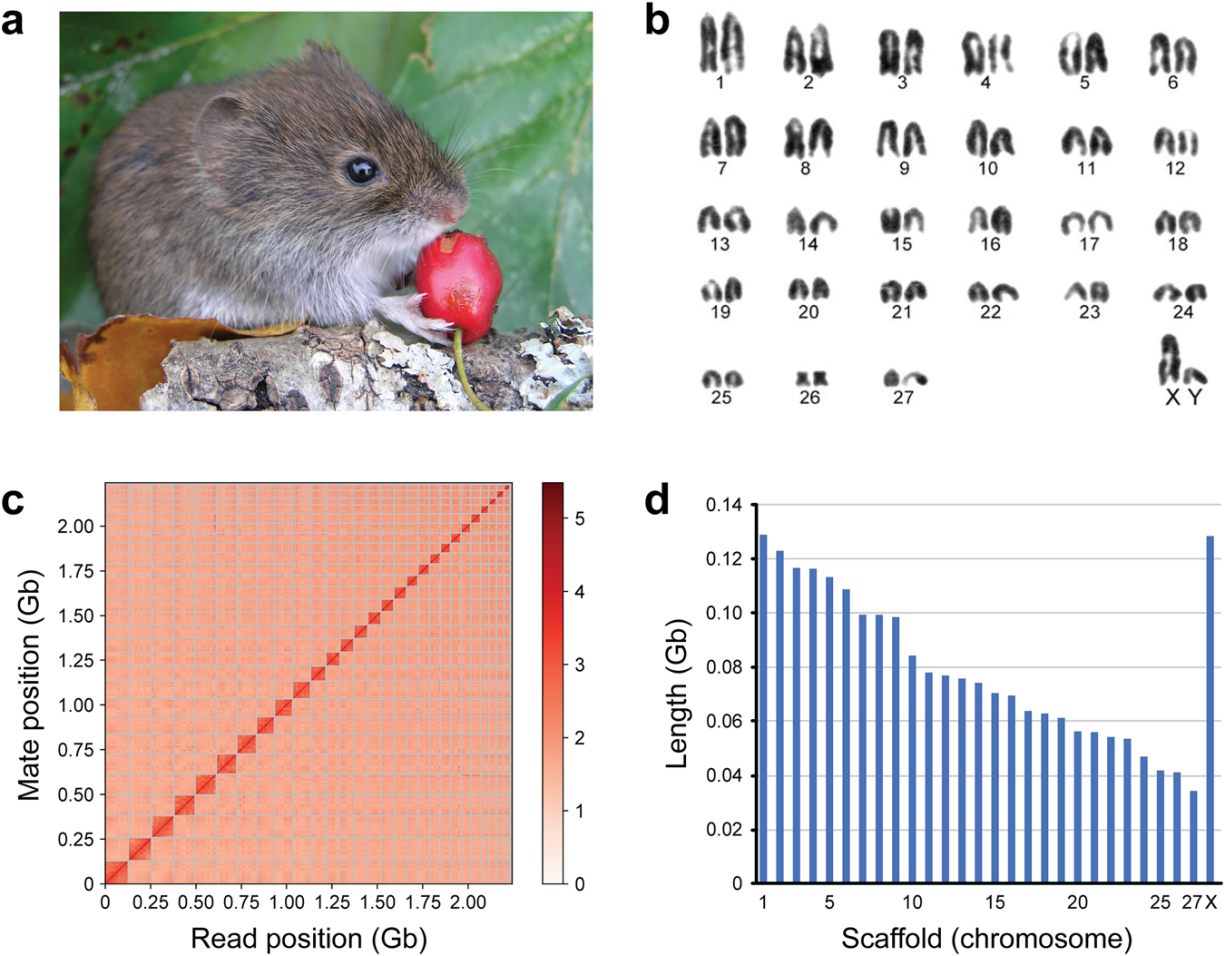
Chromosomal organization of the bank vole genome. (**a**) Photograph of a bank vole (*Clethrionomys glareolus*). Photo credit: Petr Kotlík. (**b**) Karyotype of the bank vole (2n = 56), showing 27 autosomal pairs and the X and Y sex chromosomes as described for the species, reproduced from Arslan & Zima (2014)^29^ with permission from the *Journal of Vertebrate Biology*. (**c**) Linked-density histogram of the 28 chromosome-scale scaffolds. (**d**) Length distribution of these 28 scaffolds, representing the 27 autosomes and the X chromosome present in the female individual.

The bank vole (Fig. 1a), a widespread temperate rodent of the family Cricetidae, inhabits forests and scrub habitats throughout much of Europe and western Asia. Its phylogeography, physiology and evolutionary ecology have made it a key system for understanding species responses to past and ongoing climate change in Europe^6,8^. For example, bank voles survived the Last Glacial Maximum in northern refugia beyond the traditionally recognized Mediterranean region^9–12^. It is also one of the species showing substantial lineage replacement and mixing during end-glacial colonization^13–16^. Our previous research on haemoglobin polymorphism in this species helped introduce the concept of adaptive phylogeography–an approach that links genotype–phenotype variation within species to spatial patterns of genetic structure and local adaptation^17^. The availability of a chromosome-level genome now enables these investigations to be carried out in the bank vole and related species with unprecedented resolution. Expanding such high-resolution studies across diverse taxa is essential for reconstructing the origins of present-day biodiversity and anticipating species’ responses to future climatic shifts^18,19^.

Genetic studies on the bank vole began with mitochondrial DNA (mtDNA) phylogeography, covering a range from single genes to complete mitogenomes^11,20^, and progressed to transcriptome-based SNP discovery using RNA-Seq and genotyping-by-sequencing (GBS) approaches^14,15^. Functional variation has been illustrated through integrative studies of globin gene repertoires and protein phenotypes^17^. However, the absence of a chromosome-level genome has limited insights into large-scale genome architecture, recombination and structural variation.

The bank vole’s broader significance also lies in its relevance beyond evolutionary biology. It serves as a natural reservoir for rodent-borne zoonotic parasites^21^ and pathogens, such as Puumala and other viruses^22,23^, making it an increasingly valuable system in immunology, virology and disease ecology. The availability of a chromosome-level genome allows integration of functional, ecological and fitness data with genomic variation, positioning the bank vole as a truly cross-disciplinary model^24^.

A draft genome (RefSeq accession: GCF_902806735.1) previously served as a scaffold-level reference for SNP discovery and gene-based studies, including developmental biology research^25^. While valuable, its fragmented nature limited analyses requiring chromosomal context. The bank vole mitochondrial genome, which we previously sequenced^20^, has also served as a valuable benchmark for comparative studies in other species^26^, and holds particular significance as one of the first complete mammalian mtDNA genomes annotated using RNA-Seq data^27^. A preliminary version of our chromosome-level assembly–comprising 29 large scaffolds–was already applied in population genomic analyses^8^ and transcriptomic studies of hantavirus-infected bank vole cells^28^. The finalized assembly, presented here for the first time, consists of 28 large scaffolds, each corresponding to a chromosome and consistent with the known bank vole karyotype^29^ (Fig. 1b). This chromosome-level resolution now enables detailed exploration of genome architecture, structural variation and signatures of selection–unlocking new opportunities for evolutionary, ecological and biomedical research in this increasingly important model species.

Here, we present the first comprehensive description of the finalized chromosome-level bank vole genome^30^ generated by Dovetail Genomics (Scotts Valley, CA) using 793.8 million read pairs from the short-insert library, 409 million from the Chicago library, and 404 million from the Dovetail Hi-C library. The initial *de novo* Meraculous^31^ assembly comprised 122,755 scaffolds with a total length of 2,229.96 Mb (Table 1). This draft assembly was subsequently refined using Chicago and Hi-C data with the Dovetail HiRise scaffolding pipeline (Table 1).

**Table 1:** Assembly statistics for the bank vole genome.

Assembly	Total Length (Mb)	N50 (Mb)	L50	N90 (Mb)	L90	Scaffolds	Longest Scaffold (Mb)
*De novo* assembly	2,229.96	0.038	16,371	0.009	63,075	122,755	0.593
HiRise assembly	2,241.81	98,461	10	53,427	24	4,303	128.886

Metrics are shown for the initial *de novo* Meraculous assembly and the final HiRise assembly incorporating Chicago and Hi-C scaffolding steps.

The initial HiRise assembly consisted of 4,305 scaffolds totalling 2,241.81 Mb, with an N50 of 98.46 Mb, an N90 of 46.86 Mb, and an estimated physical coverage of 19,955 ×. Eighty-four scaffolds exceeded 10 Kb, and 29 scaffolds were over 25 Mb in length, together spanning 2,231.76 Mb–indicating near chromosome-level completeness. This version was used in previous studies^8,28^.

However, the presence of 29 large scaffolds–one more than the known haploid chromosome number (n = 28)–prompted a final reassessment of the Hi-C data in light of the expected karyotype. This reanalysis led to the identification of an unjoined boundary between two scaffolds corresponding to the same chromosome. Merging these scaffolds resulted in an updated assembly with 28 chromosome-scale scaffolds, each over 30 Mb in length (Fig. 1c,d). The final assembly (Table 1) matches the known karyotype and relative chromosome sizes of the bank vole (Fig. 1b; see also^29^), and marks a significant advancement over previous resources and opens new opportunities for high-resolution studies of genome evolution, structure and function in this key species.

**Fig. 2:**
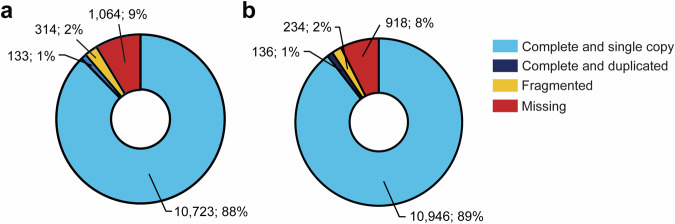
BUSCO completeness (Laurasiatheria reference set) for the initial 4,305 scaffolds (**a**), and the final 28 chromosome-level scaffolds (**b**).

BUSCO analysis^32^ (Fig. 2) conducted on the initial 4,305 scaffolds of the bank vole genome using the Laurasiatheria dataset recovered a total of 10,856 (88.7%) out of 12,234 loci. Of these, 10,723 (87.7%) were complete and single-copy, 133 (1.1%) were complete and duplicated, 314 (2.6%) were fragmented and 1,064 (8.7%) were missing (Fig. 2a). These results improved slightly in the final assembly, which consolidated the genome into 28 scaffolds and recovered 11,082 loci (90.6%), including 10,946 (89.5%) complete and single-copy BUSCO genes (Fig. 2b).

In GAWN analyses, transcriptome sequences of the bank vole^33^ were first mapped to the 28 scaffolds to predict gene structures based on homology-supported evidence. A subsequent BLASTX search aligned the predicted genes with the Swiss-Prot database^34,35^. In total, 40,392 gene loci were predicted, of which 21,029 showed homology to Swiss-Prot proteins and were therefore classified as putative protein-coding genes. The remaining loci likely represent non–protein-coding gene predictions, including non-coding RNAs, pseudogenes, partial gene models, or genes not yet represented in Swiss-Prot^36^. This distribution is comparable to the annotated mouse (M11; GRCm38.p4) and rat (GCF_015227675.2) genomes, which encode 48,709 and 42,049 gene loci, respectively, including 22,018 and 21,990 protein-coding genes^37–39^. Most bank vole protein-coding genes (19,214; 91%) show similarity to mouse (14,921) and rat (4,293) proteins in the Swiss-Prot collection. All genes (14,051) previously identified in the bank vole transcriptome assembly^33^ and matched to ENSEMBL mouse transcripts were successfully located in the genome.

**Fig. 3:**
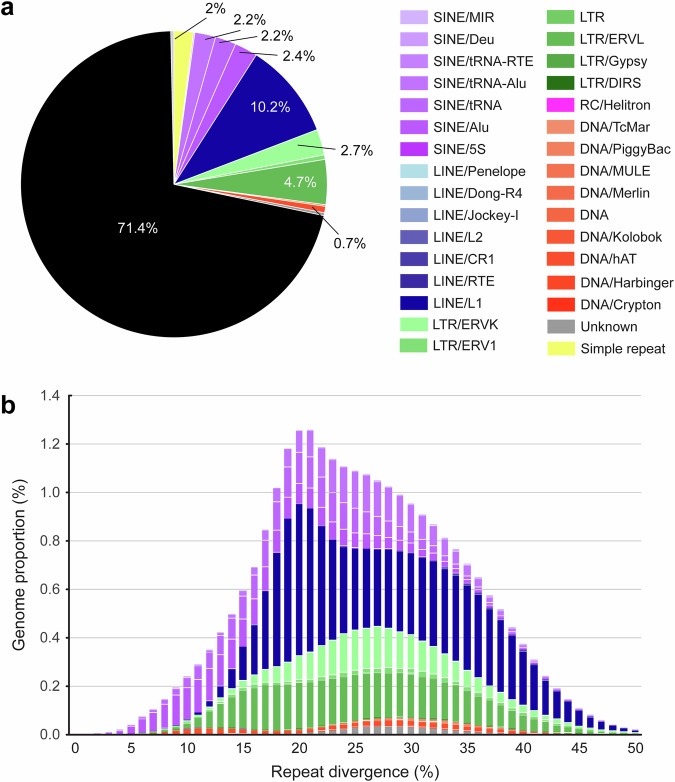
Interspersed repeat landscape of the bank vole genome. (**a**) Genome occupancy of major repeat classes as identified by RepeatMasker; the black segment represents sequence not annotated as repetitive by RepeatMasker. (**b**). Divergence landscape showing the relative age and abundance of repeat families; colours correspond to the repeat classes shown in panel (**a**). Divergence values are calculated as CpG-adjusted Kimura 2-parameter distances between repeat copies and their consensus sequences.

RepeatMasker identified repetitive sequences comprising 28.97% (646,598,510 bp) of the bank vole genome^40^. Among transposable elements, retroelements made up 25.25%, with long interspersed nuclear elements (LINEs) accounting for 10.29%, short interspersed nuclear elements (SINEs) for 7.03% and long terminal repeat (LTR) elements for 7.93% (Fig. 3a). DNA transposons constituted 0.87% of the genome. These values are comparable to the repetitive content reported in other cricetid rodent genomes^41^, although slightly lower overall (Table 2). This difference may reflect genuine species-specific variation in transposable element content, but it may also arise from differences in the versions of repeat annotation tools used, as well as from variation in assembly strategies and contiguity. Nonetheless, the repeat content shows no indication of major underrepresentation of particular repeat classes, and the overall repeat proportion differs only marginally from that reported for related rodent genomes. The repeat landscape reveals a dominant ~20% divergence peak driven by ancient SINE and LINE activity, along with a smaller, earlier signal corresponding to a modest LTR expansion (Fig. 3b).

**Table 2:** Proportion (%) of major repetitive element classes in the bank vole genome and in other cricetid rodent chromosome-level assemblies.

Species	SINEs	LINEs	LTRs	DNA Elements	Unclass.	Satellites	Simple repeats	Low compl.	Total
***Clethrionomys glareolus***	**7.03**	**10.29**	**7.93**	**0.87**	**0.61**	**0.14**	**1.75**	**0.30**	**28.97**
*Peromyscus attwateri*	9.36	11.53	10.74	1.39	0.5	0.08	2.17	0.29	36.16
*Peromyscus aztecus*	9.25	11.06	10.44	1.38	0.48	0.08	2.08	0.29	35.14
*Peromyscus eremicus*	9.22	10.54	10.51	1.45	0.46	0.08	2.07	0.28	34.68
*Peromyscus leucopus*	9.42	11.44	11.28	1.41	0.47	0.09	2.24	0.31	36.73
*Peromyscus maniculatus*	9.04	10.44	10.33	1.38	0.47	0.07	2.11	0.28	34.19
*Peromyscus melanophrys*	9.31	11.31	10.72	1.38	0.49	0.09	2.29	0.29	35.95
*Peromyscus nudipes*	9.3	11.37	10.73	1.39	0.49	0.08	2.05	0.28	35.78
*Peromyscus polionotus*	8.17	9.55	9.42	1.3	0.43	0.06	1.91	0.26	31.16

Abbreviations: Unclass., unclassified repeats; Low compl., low-complexity repeats.

Finally, GenMap^42^ was used to complement the RepeatMasker-based repeat annotation by identifying regions of low mappability, which typically correspond to low-complexity or repetitive sequence. Mappability was calculated using 100-bp k-mers allowing up to two mismatches, and regions with low mappability were interpreted as putative repetitive elements not fully captured by RepeatMasker. In total, 20,062,853 GenMap-identified sites were masked^40^. The final masked genome represents the union of RepeatMasker- and GenMap-based masking, generating a more comprehensively masked version of the genome to improve the accuracy of short-read mapping in downstream analyses^40^.

## Methods

### Specimen collection and preparation

The genome sequenced here belongs to a female bank vole captured near Želízy (Czech Republic). Liver, spleen, kidney, lung and heart tissues, along with 500 μl of blood, were collected, flash-frozen in liquid nitrogen and stored at −80°C. High molecular weight (HMW) genomic DNA was extracted using the Qiagen Genomic DNA Extraction Kit (Qiagen, Valencia, CA). Subsequently, the samples were processed for sequencing and genome assembly by Dovetail Genomics (Scotts Valley, CA), with a *de novo* shotgun assembly scaffolded using Chicago and Hi-C libraries and the HiRise software pipeline^43^.

### *De Novo* assembly of the bank vole genome

A *de novo* assembly was generated by using a combination of two paired-end libraries (mean insert size of ~350 bp). *De novo* assembly was performed using Meraculous^31^ with a kmer size of 73. The input dataset comprised 793.8 million read pairs obtained from paired-end libraries, totalling 238.1 Gb. Prior to assembly, reads were trimmed for quality, sequencing adaptors and mate pair adaptors using Trimmomatic^44^.

### Preparation and sequencing of Chicago libraries

Three Chicago libraries were constructed as previously described^43^. Briefly, approximately 500 ng HMW gDNA (with a mean fragment length of 100 kb) was *in vitro* reconstituted into chromatin and fixed with formaldehyde for each library. Subsequently, the fixed chromatin was digested with DpnII and the resulting 5′ overhangs were biotinylated before ligation of the free blunt ends. Following ligation, crosslinks were reversed and the DNA was purified from protein. The purified DNA underwent a biotin removal step to eliminate any residual biotin that was not present within the ligated fragments. The DNA was then sheared to a mean fragment size of around 350 bp and sequencing libraries were constructed using NEBNext Ultra enzymes (NEB, Ipswich, MA, USA) and Illumina-compatible adapters. Biotin-containing fragments were isolated with streptavidin beads prior to PCR enrichment of each library. Libraries were then sequenced on an Illumina HiSeq X platform with a 2 × 150 bp read length. The number of read pairs generated for each library were as follows: 127 million for library 1; 130 million for library 2; and 152 million for library 3. Together, the Chicago library reads provided a 57.42-fold physical coverage of the genome (1–100 kb pairs). Physical coverage values were calculated as the number of read pairs with insert sizes between 1 and 100 kb that span each position in the input assembly.

### Preparation and sequencing of Dovetail Hi-C libraries

Three Dovetail Hi-C libraries were prepared in a similar manner as described previously^45^. In brief, for each library, chromatin within the nucleus was first fixed with formaldehyde and then extracted. The fixed chromatin was then processed and the libraries were prepared and sequenced using a same approach as described for the Chicago libraries. The number of read pairs generated for each Hi-C library were as follows: 124 million for library 1; 151 million for library 2; and 129 million for library 3. Together, the reads from these Dovetail Hi-C libraries provided a 19,955.19 × physical coverage of the genome (10–10,000 kb pairs). The physical coverage was determined by counting the number of read pairs with insert sizes between 10 and 10,000 kb that spanned each position in the input assembly. Physical coverage reflects the long-range span of read pairs and provides the genomic connectivity needed for accurate scaffolding and long-range structural correctness of the assembly.

### Scaffolding of the assembly with HiRise

The *de novo* assembly, shotgun reads, Chicago library reads and Dovetail Hi-C library reads were all used as input data for the HiRise pipeline, which was designed to use proximity ligation data to scaffold genome assemblies^43^. Subsequently, an iterative analysis was performed. First, sequences from the shotgun and Chicago libraries were aligned to the draft input assembly using a modified SNAP read mapper (http://snap.cs.berkeley.edu). The separations of the mapped Chicago read pairs were analysed by HiRise to create a likelihood model for the genomic distance between read pairs. This model was then used to identify and break putative misjoins, to score prospective joins and create joins above a threshold. After alignment and scaffolding of the Chicago data, sequences from the Dovetail Hi-C library were aligned and scaffolded using the same approach. After scaffolding, the shotgun sequences were used to fill gaps between contigs.

The initial assembly of 4,620 scaffolds was refined using the HiRise pipeline, resulting in 4,305 scaffolds with a total length of 2,241.81 Mb. Of these, 84 scaffolds exceeded 10 kb in length and 29 were longer than 25 Mb, collectively spanning 2,231.76 Mb – approaching chromosome-level completeness, though still exceeding the expected haploid chromosome number (n = 28) by one scaffold. To resolve this, an additional round of HiRise scaffolding was performed, in which the existing Hi-C data were reanalysed to generate refined contact maps and reassess scaffold joins.

### Genome quality assessment and annotation

We evaluated genome completeness using Benchmarking Universal Single-Copy Orthologs (BUSCO) version 5.4.7^46^. For this assessment, we employed the reference gene set of laurasiatheria_odb10 (12,234 loci in total) from the OrthoDB database of orthologs (www.orthodb.org) and ran the program with default parameters.

A transcriptome assembly from bank vole heart^33^ was then used to annotate the genome using the Genome Annotation Without Nightmares (GAWN) version 0.3.2 pipeline (https://github.com/enormandeau/gawn). The pipeline utilized GMAP^47^ to map transcriptome sequences to the genome assembly, generating a gff3 file and retrieved annotations from the Swiss-Prot database^34^ (https://www.uniprot.org/uniprotkb) using blastplus utilities (blastx) version 2.12.0 +^48^.

### Repetitive element annotation and mappability calculation

To identify, classify and mask repetitive elements, including low-complexity sequences and interspersed repeats, we used RepeatMasker version 4.1.2 (https://www.repeatmasker.org/) in conjunction with RMBlast search engine version 2.11.0 + . RepeatMasker^49^ was utilized to scan the bank vole genome for rodent repetitive sequences from Dfam version 3.3, an open-access database of families of repetitive DNA elements^50^. To calculate mappability scores of the bank vole genome, we used GenMap v.1.3.0 software^42^ with the options “-K 100” and “-E 2”. Subsequently, we masked sites with a mappability score less than 1 in the bank vole genome. These analyses were performed to provide masked genome versions for downstream applications.

## Data Records

The raw sequencing data have been deposited in the NCBI Sequence Read Archive under BioProject PRJNA1005562, within the SRA Study SRP650011^51^. The final genome assembly of the bank vole genome was deposited at GenBank (accession GCA_055000105.1)^30^. Genome annotation files (gff3 file and annotation table), as well as the RepeatMasker- and GenMap-masked genome assembly files, are available in the Figshare repository^40^.

## Technical Validation

The 2.24 Gb bank vole genome assembly is resolved into 28 chromosome-scale scaffolds, consistent with the expected haploid chromosome number (n = 28) as supported by karyotype analyses^29^ (Fig. 1b–d). The assembly shows high completeness (90.6%) based on the Laurasiatheria BUSCO set (Fig. 2). Genome annotation successfully identified 21,029 protein-coding genes with functional annotation, a number comparable to those reported for the mouse (22,018) and rat (21,990) genomes^37–39^. The repetitive landscape of the bank vole genome is broadly consistent with that of other cricetid rodents (Table 2). This high-quality assembly provides a robust foundation for evolutionary and ecological genomics in this species, as well as for studies of rodent-borne zoonotic parasites and pathogens. Continued advances in long-read sequencing technologies and assembly algorithms are expected to further improve genome completeness and enable deeper insights into gene family evolution, paralogous relationships, and structural variation.

## Data Availability

Raw paired-end Illumina sequencing reads are available from the NCBI Sequence Read Archive under BioProject PRJNA1005562 (SRA Study SRP650011)^51^. The genome assembly of the bank vole is deposited in NCBI GenBank under accession GCA_055000105.1^30^. Genome annotation data and masked genome assemblies are available from Figshare (10.6084/m9.figshare.29329526)^40^. This dataset includes a genome annotation file in GFF3 format, a corresponding tab-delimited annotation table, and three masked genome assembly FASTA files: one with repetitive elements masked using RepeatMasker, one with regions of low sequence mappability masked using GenMap, and one masked for both repetitive elements and low-mappability regions.

## Code availability

All data analyses were performed with bioinformatics software describe in Methods. No custom script or code were utilized in this study.

## References

[1] Ellegren, H. Genome sequencing and population genomics in non-model organisms. *Trends Ecol. Evol.* **29**, 51–63 (2014). 24139972 10.1016/j.tree.2013.09.008

[2] da Fonseca, R. R. *et al*. Next-generation biology: Sequencing and data analysis approaches for non-model organisms. *Mar. Genomics* **30**, 3–13 (2016). 27184710 10.1016/j.margen.2016.04.012

[3] Etherington, G. J. *et al*. Sequencing smart: De novo sequencing and assembly approaches for a non-model mammal. *Gigascience* **9**, 1–14 (2020). 10.1093/gigascience/giaa045 PMC7216774 32396200

[4] Russell, J. J. *et al*. Non-model model organisms. *BMC Biol.* **15**, 1–31 (2017). 28662661 10.1186/s12915-017-0391-5 PMC5492503

[5] Weiskopf, S. R. *et al*. Climate change effects on biodiversity, ecosystems, ecosystem services, and natural resource management in the United States. *Sci. Total Environ.* **733**, 137782 (2020). 32209235 10.1016/j.scitotenv.2020.137782

[6] Kotlík, P., Marková, S., Horníková, M., Escalante, M. A. & Searle, J. B. The bank vole (*Clethrionomys glareolus*) as a model system for adaptive phylogeography in the European theater. *Front. Ecol. Evol.* **10**, 866605 (2022).

[7] Kryštufek, B. *et al*. Back to the future: the proper name for red-backed voles is *Clethrionomys* Tilesius and not *Myodes* Pallas. *Mammalia* **84**, 214–217 (2020).

[8] Marková, S. *et al*. Local adaptation and future climate vulnerability in a wild rodent. *Nat. Commun.* **14**, 7480 (2023). 38030627 10.1038/s41467-023-43383-z PMC10686993

[9] Bilton, D. T. *et al*. Mediterranean Europe as an area of endemism for small mammals rather than a source for northwards postglacial colonization. *Proc. R. Soc. B Biol. Sci.* **265**, 1219–1226 (1998). 10.1098/rspb.1998.0423 PMC1689182 9699314

[10] Deffontaine, V. *et al*. Beyond the Mediterranean peninsulas: evidence of central European glacial refugia for a temperate forest mammal species, the bank vole (*Clethrionomys glareolus*). *Mol. Ecol.* **14**, 1727–1739 (2005). 15836645 10.1111/j.1365-294X.2005.02506.x

[11] Kotlík, P. *et al*. A northern glacial refugium for bank voles (*Clethrionomys glareolus*). *Proc. Natl. Acad. Sci.* **103**, 14860–14864 (2006). 17001012 10.1073/pnas.0603237103 PMC1595441

[12] Bhagwat, S. A. & Willis, K. J. Species persistence in northerly glacial refugia of Europe: a matter of chance or biogeographical traits? *J. Biogeogr.* **35**, 464–482 (2008).

[13] Searle, J. B. *et al*. The Celtic fringe of Britain: insights from small mammal phylogeography. *Proc. R. Soc. B Biol. Sci.* **276**, 4287–4294 (2009). 10.1098/rspb.2009.1422 PMC2817114 19793757

[14] Kotlík, P., Marková, S., Konczal, M., Babik, W. & Searle, J. B. Genomics of end-Pleistocene population replacement in a small mammal. *Proc. R. Soc. B Biol. Sci.* **285**, 20172624 (2018). 10.1098/rspb.2017.2624 PMC5829201 29436497

[15] Marková, S. *et al*. High genomic diversity in the bank vole at the northern apex of a range expansion: The role of multiple colonizations and end-glacial refugia. *Mol. Ecol.* **29**, 1730–1744 (2020). 32248595 10.1111/mec.15427

[16] Horníková, M., Marková, S., Lanier, H. C., Searle, J. B. & Kotlík, P. A dynamic history of admixture from Mediterranean and Carpathian glacial refugia drives genomic diversity in the bank vole. *Ecol. Evol.* **11**, 8215–8225 (2021). 34188881 10.1002/ece3.7652 PMC8216894

[17] Kotlík, P. *et al*. Adaptive phylogeography: Functional divergence between haemoglobins derived from different glacial refugia in the bank vole. *Proc. R. Soc. B Biol. Sci.* **281**, 20140021 (2014). 10.1098/rspb.2014.0021 PMC4046400 24827438

[18] Tian, L. & Benton, M. J. Predicting biotic responses to future climate warming with classic ecogeographic rules. *Curr. Biol.* **30**, R744–R749 (2020). 32634410 10.1016/j.cub.2020.06.003

[19] Escalante, M. A., Marková, S., Searle, J. B. & Kotlík, P. Genic distribution modelling predicts adaptation of the bank vole to climate change. *Commun. Biol.* **5**, 981 (2022). 36114276 10.1038/s42003-022-03935-3 PMC9481625

[20] Bendová, K., Marková, S., Searle, J. B. & Kotlík, P. The complete mitochondrial genome of the bank vole *Clethrionomys glareolus* (Rodentia: Arvicolinae). *Mitochondrial DNA* **27**, 111–112 (2016). 24438307 10.3109/19401736.2013.873927

[21] McManus, A., Holland, C. V., Henttonen, H. & Stuart, P. The invasive bank vole (*Myodes glareolus*): A model system for studying parasites and ecoimmunology during a biological invasion. *Animals* **11**, 2529 (2021). 34573495 10.3390/ani11092529 PMC8464959

[22] Ermonval, M., Baychelier, F. & Tordo, N. What do we know about how hantaviruses interact with their different hosts? *Viruses* **8**, 223 (2016). 27529272 10.3390/v8080223 PMC4997585

[23] Rossi, C. *et al*. Evolutionary relationships of Ljungan virus variants circulating in multi-host systems across Europe. *Viruses* **13**, 1317 (2021). 34372523 10.3390/v13071317 PMC8310206

[24] Górska, J. *et al*. Beyond the laboratory: the bank vole (*Clethrionomys glareolus*) as a novel model organism in biological research. *Front. Zool.* **22**, 26 (2025). 41029728 10.1186/s12983-025-00578-y PMC12487395

[25] Calamari, Z. T. *et al*. Bank vole genomics links determinate and indeterminate growth of teeth. *BMC Genomics* **25**, 1000 (2024). 39472825 10.1186/s12864-024-10901-2 PMC11523675

[26] Donath, A. *et al*. Improved annotation of protein-coding genes boundaries in metazoan mitochondrial genomes. *Nucleic Acids Res.* **47**, 10543 (2019). 31584075 10.1093/nar/gkz833 PMC6847864

[27] Marková, S., Filipi, K., Searle, J. B. & Kotlík, P. Mapping 3’ transcript ends in the bank vole (*Clethrionomys glareolus*) mitochondrial genome with RNA-Seq. *BMC Genomics* **16**, 870 (2015). 26503603 10.1186/s12864-015-2103-2 PMC4624183

[28] Gallo, G. *et al*. Diverse susceptibilities and responses of human and rodent cells to orthohantavirus infection reveal different levels of cellular restriction. *PLoS Negl. Trop. Dis.* **16**, e0010844 (2022). 36223391 10.1371/journal.pntd.0010844 PMC9591050

[29] Arslan, A. & Zima, J. Karyotypes of the mammals of Turkey and neighbouring regions: A review. *Folia Zool.* **63**, 1–62 (2014).

[30] Marková, S., White, T. A., Searle, J. B. & Kotlík, P. Chromosome-level genome of the bank vole (*Clethrionomys glareolus*): a resource for eco-evo-disease research. *GenBank* https://identifiers.org/ncbi/insdc.gca:GCA_055000105.1 (2026). 10.1038/s41597-026-06924-x PMC13057007 41748635

[31] Chapman, J. A. *et al*. Meraculous: De novo genome assembly with short paired-end reads. *PLoS One* **6**, e23501 (2011). 21876754 10.1371/journal.pone.0023501 PMC3158087

[32] Manni, M., Berkeley, M. R., Seppey, M. & Zdobnov, E. M. BUSCO: assessing genomic data quality and beyond. *Curr. Protoc.* **1**, e323 (2021). 34936221 10.1002/cpz1.323

[33] Babik, W. *et al*. Heart transcriptome of the bank vole (*Myodes glareolus*): Towards understanding the evolutionary variation in metabolic rate. *BMC Genomics* **11**, 390 (2010). 20565972 10.1186/1471-2164-11-390 PMC2996923

[34] The UniProt Consortium. UniProt: the Universal Protein Knowledgebase in 2023. *Nucleic Acid Res.* **51**, D523–D531 (2023). 36408920 10.1093/nar/gkac1052 PMC9825514

[35] MacDougall, A. *et al*. UniRule: A unified rule resource for automatic annotation in the UniProt knowledgebase. *Bioinformatics* **36**, 4643–4648 (2020). 32399560 10.1093/bioinformatics/btaa485 PMC7750954

[36] Frankish, A. *et al*. GENCODE: reference annotation for the human and mouse genomes in 2023. *Nucleic Acids Res.* **51**, D942–D949 (2023). 36420896 10.1093/nar/gkac1071 PMC9825462

[37] Breschi, A., Gingeras, T. R. & Guigó, R. Comparative transcriptomics in human and mouse. *Nat. Rev. Genet.* **18**, 425–440 (2017). 28479595 10.1038/nrg.2017.19 PMC6413734

[38] Frankish, A. *et al*. GENCODE reference annotation for the human and mouse genomes. *Nucleic Acids Res.* **47**, D766–D773 (2019). 30357393 10.1093/nar/gky955 PMC6323946

[39] Vedi, M. *et al*. 2022 updates to the Rat Genome Database: A Findable, Accessible, Interoperable, and Reusable (FAIR) resource. *Genetics* **224**, iyad042 (2023). 36930729 10.1093/genetics/iyad042 PMC10474928

[40] Marková, S., White, T. A., Searle, J. B. & Kotlík, P. Chromosome-level genome of the bank vole (*Clethrionomys glareolus*): a resource for eco-evo-disease research. *figshare*10.6084/m9.figshare.29329526 (2026). 10.1038/s41597-026-06924-x PMC13057007 41748635

[41] Tigano, A., Colella, J. P. & MacManes, M. D. Comparative and population genomics approaches reveal the basis of adaptation to deserts in a small rodent. *Mol. Ecol.* **29**, 1300–1314 (2020). 32130752 10.1111/mec.15401 PMC7204510

[42] Pockrandt, C., Alzamel, M., Iliopoulos, C. S. & Reinert, K. GenMap: Ultra-fast computation of genome mappability. *Bioinformatics* **36**, 3687–3692 (2020). 32246826 10.1093/bioinformatics/btaa222 PMC7320602

[43] Putnam, N. H. *et al*. Chromosome-scale shotgun assembly using an *in vitro* method for long-range linkage. *Genome Res.* **26**, 342 (2016). 26848124 10.1101/gr.193474.115 PMC4772016

[44] Bolger, A. M., Lohse, M. & Usadel, B. Trimmomatic: a flexible trimmer for Illumina sequence data. *Bioinformatics* **30**, 2114 (2014). 24695404 10.1093/bioinformatics/btu170 PMC4103590

[45] Lieberman-Aiden, E. *et al*. Comprehensive mapping of long-range interactions reveals folding principles of the human genome. *Science* **326**, 289–293 (2009). 19815776 10.1126/science.1181369 PMC2858594

[46] Simão, F. A., Waterhouse, R. M., Ioannidis, P., Kriventseva, E. V. & Zdobnov, E. M. BUSCO: Assessing genome assembly and annotation completeness with single-copy orthologs. *Bioinformatics* **31**, 3210–3212 (2015). 26059717 10.1093/bioinformatics/btv351

[47] Wu, T. D. & Watanabe, C. K. GMAP: A genomic mapping and alignment program for mRNA and EST sequences. *Bioinformatics* **21**, 1859–1875 (2005). 15728110 10.1093/bioinformatics/bti310

[48] Camacho, C. *et al*. BLAST+: architecture and applications. *BMC Bioinformatics* **10**, 421 (2009). 20003500 10.1186/1471-2105-10-421 PMC2803857

[49] Smit, A. F., Hubley, R. & Green, P. RepeatMasker Open-4.0. Available at: http://www.repeatmasker.org.

[50] Bao, W., Kojima, K. K. & Kohany, O. Repbase Update, a database of repetitive elements in eukaryotic genomes. *Mob. DNA* **6**, 11 (2015). 26045719 10.1186/s13100-015-0041-9 PMC4455052

[51] *NCBI Sequence Read Archive.* https://identifiers.org/ncbi/insdc.sra:SRP650011 (2025).

